# Polyethylene/Polyamide Blends Made of Waste with Compatibilizer: Processing, Morphology, Rheological and Thermo-Mechanical Behavior

**DOI:** 10.3390/polym13142385

**Published:** 2021-07-20

**Authors:** Dorota Czarnecka-Komorowska, Jagoda Nowak-Grzebyta, Katarzyna Gawdzińska, Olga Mysiukiewicz, Małgorzata Tomasik

**Affiliations:** 1 Institute of Materials Technology, Poznan University of Technology, 60-965 Poznan, Poland; olga.mysiukiewicz@put.poznan.pl; 2 Faculty of Mechanical Engineering, Poznan University of Technology, 60-965 Poznan, Poland; jagoda.pa.nowak@doctorate.put.poznan.pl; 3 Department of Machines Construction and Materials, Maritime University of Szczecin, 71-650 Szczecin, Poland; k.gawdzinska@am.szczecin.pl; 4 Department of Interdisciplinary Dentistry, Pomeranian Medical University, 70-111 Szczecin, Poland; malgorzata.tomasik@pum.edu.pl

**Keywords:** recycling, plastics waste, extrusion, polyethylene/polyamide blends, compatibility, physical properties, structure

## Abstract

The aim of this study was to develop a polyethylene/polyamide (R-PE/R-PA) regranulated product made from post-consumer wastes grafted with polyethylene-graft-maleic anhydride (PE-g-MAH) by reactive extrusion in a twin-screw extruder equipped with an external mixing zone. The compatibility effect of PE-g-MAH used as a modifier in R-PE/R-PA blends was evaluated by means of differential scanning calorimetry (DSC) and dynamic mechanical thermal analysis (DMTA), while the analysis of the chemical structure of this blend was carried out by Fourier transform infrared spectroscopy (FT-IR). The thermal properties, complex viscosity, and selected usage properties of R-PE/R-PA blends compatibilized with PE-g-MAH, i.e., density and water absorption, were evaluated. The morphology of the blends with and without the compatibilizer was observed by scanning electron microscopy. The R-PE/R-PA/MAH blend shows heterogenic structure, which is a result of the chemical reaction in reactive extrusion between functional groups of PE-g-MAH used as modifier and the end groups of R-PA6. The results show that the R-PE/R-PA blend with increased PE-g-MAH content showed increased hardness, stiffness, and ultimate tensile strength due to the increased degree of crystallinity. The increase in crystallinity is proportional to the improvement of the mechanical properties. Moreover, it is shown that 1 wt.% PE-g-MAH added to the R-PE/R-PA waste blend increases the interfacial interactions and compatibility between R-PE and R-PA, resulting in decreased polyamide particle size. Finally, the results show that it is possible to produce good quality regranulated products with advantageous properties and structure from immiscible polymer waste for industrial applications.

## 1. Introduction

Nowadays, polymer blends based on waste, from which new materials with tailor-made properties are made, are very popular among researchers and could be beneficial both from an environmental and economical point of view. Due to the change of the traditional model of the linear economy “take–make–dispose” on the circular model is considered [1]. The linear model represents the one-way consumption of plastic products, which results in the production of large amounts of waste materials [2]. Disposing of this waste in an unprocessed form results in an endless need to extract non-renewable raw materials for use in the production of polymer materials [3]. Consequently, this leads to the overexploitation of natural resources, increased greenhouse gas emissions, and environmental degradation [4,5]. An alternative solution is to introduce a circular economy [6], which assumes an increased level of raw material recovery from waste through material recycling, thus reducing the total greenhouse gas emissions [5]. The circular economy works to ensure that the value of materials, and products are preserved for as long as possible while minimizing waste [6]. The efficient processing of polymer waste remains a difficult challenge, and the recycling process is still the best way to manage wastes [7]. Many researchers are working on developing new technologies for recycling polymers [8,9,10]. They are also developing new recycled materials, which, when properly modified, can function as full-value and cheap “secondary” raw materials for plastic products that can be used in manufacturing processes either instead of or with virgin raw materials. The ideal materials that meet both economic and environmental criteria that can be used in mechanical engineering are recycled polymer regranulates of various engineering plastics, i.e., polyethylene (PE), polyamide (PA), polypropylene (PP), and polyoxymethylene (POM) [11,12]. Polymer regranulates, as a rule, have reduced mechanical properties and thermal resistance, and many procedures can be used to improve these properties, such as mixing with virgin polymers; adding fibrous fillers, i.e., glass fibers, and natural fibers; and modifying with powder fillers such as basalt [13], talc [14], polysilsesqioxanes (POSS) [15,16], or montmorillonite (MMT) [17].

Polyethylene is one of the commercial plastics widely used in packaging applications such as films and bottles, etc., which is equivalent to generating a lot of amounts of post-consumer waste [9]. Among the recycling methods, blending recycled polyethylene with other polymers is very attractive because of the ability to combine different material properties. For example, the use of polyamide can improve the stiffness and strength of materials. Unfortunately, but the above-mentioned PE/PA blending obtained by a simple blending technique shows reduced strength properties than polyethylene or polyamide due to poor compatibility between PE and PA. One way to improve the properties of blends can be to incorporate compatibilizers into immiscible polymers, such as polyethylene with polyamide in the molten state by extrusion [18]. 

In the case of waste polymer blends, the problem is much more serious, as they are often thermodynamically non-compatible blends with low or no adhesion [9]. This leads to the production of regranulates with unfavorable mechanical and processing properties. Improving the interfacial adhesion can be accomplished by chemical compatibilization by introducing a compatibilizer, an additional third component that is miscible with both phases [19,20] and copolymer whose one part is miscible with one phase and another with another phase [21] and or by reactive processing [20,21], i.e., reactive formation of graft, block, or lightly crosslinked copolymer, formation of ionically bonded; structures trans-reactions, and mechano-chemical blending [20]. The compatibilization must accomplish optimization of the interfacial tension, stabilize the morphology against high stresses during enhance adhesion between the phases in the solid state [20]. 

The most frequently used compatibilizers in the case of polymer mixtures are ethylene copolymers intended for the processing of waste polypropylene (PP) and polyethylene (PE) blends, lattices (S-E-B) for styrene blends, and maleic anhydride, especially in the case of polyolefins recyclates. For instance, Janik et al. [22] studied the improvement of mechanical properties such as tensile and flexural strength, and modulus, and elongation at break of polycarbonate/polypropylene mixtures using polypropylene-graft-maleic anhydride (PP-g-MA). Similarly, Mengual et al. [23] researched the use of PP-g-MA and polyethylene-graft-maleic anhydride (PE-g-MA) or styrene-ethylene/butylene-styrene-graft-maleic anhydride (SEBS-g-MA) for polymer modification. Three types of application were presented: improving the properties of the base compound, as a compatibilizer in the blending of two materials, and as an additional component in the mixing of various materials [23]. 

Moreover, in the last years, many papers have been done to improve the compatibility of PE/PA blends [24,25,26,27,28,29]. For instance, Jeziórska et al. [24] studied the influence of polyethylene grafted with maleic anhydride (PE-g-MAH) on the mechanical and structural properties of polyamide (PA) and textile wastes containing poly(ethylene terephthalate) (PET). The chemical reactions of PE-g-MAH with blended components were studied by Fourier transform infrared spectroscopy (FT-IR) [24]. Jeziórska at al. [25] also reported that the incorporation of ricinol-2-oxazoline methylmaleate (MRO) in PA6/LDPE blends leads to an increase in the elastic modulus and impact strength of the blends. The PA6/LDPE/MRO blends show heterogenic structure, which, as a result of chemical reactions going during the extrusion process, stabilizes at a microphase level probably of functional groups of PA with oxazoline groups grafted onto LDPE [25]. A similar effect was observed in case of polyamide 6 and polyolefins blends functionalized with acrylic acid (polyethylene—PE–AA and polypropylene—PP–AA) by Psarski et al. [26].

Wahab et al. [27] investigated the compatibilization effects of PE-g-MA on mechanical, thermal and swelling properties of high density polyethylene (HDPE)/natural rubber (NR)/thermoplastic tapioca starch (TPS) blends, and found the effectiveness of PE-g-MA as compatibilizer in improving the miscibility between HDPE/NR/TPS blends. 

Majid et al. [28] studied the effects of PE-g-MA on the morphology, water absorption tensile and mechanical properties of LDPE/thermoplastic sago starch (TPSS) blends and found that PE-g-MA acted as an effective compatibilizer for the LDPE/TPSS blends. All these studies confirmed the beneficial effect of compatibilizers on the structure, especially the even distribution of components and selected functional properties of blends. 

As described by Huitric et al. [29], the addition of a compatibilizer promotes good dispersion and structure stabilization by suppressing coalescence to increase interfacial adhesion. The compatibilizing elements can include diblock copolymers, mixed with both phases, or block or graft copolymers formed by the interface reaction during mixing. The copolymer (acting on the surface of the droplets) inhibits coalescence very well by lowering the interfacial tension, which facilitates disintegration. The main result of these studies is the essential size reduction of the dispersed phase due to the suppression of coalescence. This is achieved using steric repulsion between the block copolymers present at the interface and the Gibbs–Marangoni effect induced by the local concentration of the compatibilizer’s gradient at the interface [29].

Therefore, the paper presents the development process of manufacturing R-PE/R-PA regranulates based on immiscible waste polymers. Regranulates from these materials, including polyethylene and polyamide, were modified with PE-g-MAH as compatibilizer. The obtained R-PE/R-PA/MAH regranulates are characterized by defined properties and structure, useful for industrial applications, including the production of blown films.

## 2. Experimental Sections

### 2.1. Materials and Procedures

The regranulate was prepared from two commercial plastics, low-density polyethylene and polyamide. Polyethylene and polyamide 6 from post-consumer waste and their blends, modified with polyethylene-graft-maleic anhydride (PE-g-MAH) copolymer as a compatibilizer were investigated in our research. PE-g-MA is a compatibilizer for polymer blends which serves as support for polar to nonpolar substances [23]. The compatibilizing agent PE-g-MAH (viscosity 1.700–4.500 cP at 140 °C, T_m (DSC)_ = 105 °C, density 0.9 g/cm^3^ at 25 °C) [30] was purchased from Sigma–Aldrich (St. Louis, MO, USA). Polyamide 6 (PA 6) grade wastes (trade name Tarnamid), supplied by Azoty Group S.A. (Tarnów, Poland), and low-density polyethylene (LDPE) wastes (trade name Malen), supplied by Basell Orlen Polyolefines (Płock, Poland), were obtained from a local Polish supplier. Sample codes and polymer blend compositions are detailed in Table 1. 

The components were first dried at 80 °C for 24 h using an ULE 500 cabinet dryer (Memmert GmbH + Co. KG, Schwabach, Germany). The blends, containing 80 wt.% of R-PE with different contents of R-PA (20, 19, or 17 wt.%) and PE-g-MAH (1 and 3 wt.%), were manufactured by mixing in a molten state (Table 1). The mixing and reacting of the components was performed under controlled conditions [31] using an EH-16.2D co-rotating twin screw extruder (Zamak Mercator Sp. z o.o. Skawina, Poland) with a length/diameter ratio (L/D) of 40 mm, a screw diameter (D) of 16 mm, and a capillary die diameter (d) of 8 mm operating at 100 rpm and with the following temperatures: 250–240 °C, 235 °C, 235 °C, 230 °C, and 230 °C along the barrel and 240 °C for the die. The extruded rod was air-dried and pelletized. Next, the regranulates were dried for 24 h at 80 °C. Dumbbell-shaped specimens for tensile tests were injection-molded using an Eco e-mac 50 (Engel Austria GmbH, Schwertberg, Austria) with a temperature of 230–250 °C, mold temperature of 25 °C, and 35 s cooling time.

### 2.2. Methods

#### 2.2.1. Scanning Electron Microscopy (SEM)

The morphology of the R-PE/R-PA grafted by PE-g-MAH blends was investigated with a MIRA3 scanning electron microscope (TESCAN Brno, s.r.o., Brno, Czech Republic) with high-resolution imaging. The specimens were fractured in liquid nitrogen and then coated with a thin layer of 20 μm carbon powder. The dispersion and dimension domains of R-PA particles in the R-PE matrix was investigated by scattered electron (SE) signal, with an accelerating voltage of 10 kV. A magnification of 5000× was used.

#### 2.2.2. Density and Water Absorption 

The solid mass of the R-PE, R-PA and their blends were measured by an electronic balance AXIS AD50-AD200 (AXIS, Gdansk, Poland). The density was measured based on PN-EN ISO 1183–1:2004 [32] standards using the hydrostatic method. Ethyl alcohol (analytical grade, supplied by Chempur, Piekary Śląskie, Poland) as an immersion liquid, was used, and measurements were made of 5 samples from each series.

Water absorption (WA) tests were carried out on sets of 5 samples for R-PE, R-PA, and each type of R-PE/R-PA blend in accordance with ISO 62:2008 [33]. In the test procedure, all specimens were dried in a cabinet dryer at 80 °C for 24 h, then their weights were measured. After 24 h immersion in water at 23 °C, samples were taken out of the water. The surface was dried with a cloth and weight was measured again. The percentage of water absorption by the different polymer blends was calculated with the following Equation (1):

(1) $$WA\left(\%\right)=\frac{m_{s}-m_{0}}{m_{0}}\cdot 100$$

where $m_{0}$ is the weight of the dry sample (g) and $m_{s}$ is the weight of the sample after 24 h water immersion (g).

#### 2.2.3. Melt Mass-Flow Rate (MFR)

Melt flow properties provide significant insight for thermoplastic manufacturing. The MFR is an indicator of the flow properties of the material in molten state. The MFR of the blends with and without compatibilizer was measured using a Kayeness melt flow plastometer (model LMI 4003, Dynisco, Franklin, MA, USA), according to the standard test method ISO 1133-1:2011 [34]. The measurements were conducted at a temperature of 230 °C under a 2.16 kg load. The average of 10 readings was calculated to determine the MFR.

#### 2.2.4. Melt Rheology

The rheological behavior of the R-PE/R-PA blends grafted by PE-g-MAH was evaluated using rotational rheometer in small-amplitude oscillation shearing mode. A MCR301 apparatus (Anton Paar GmbH, Graz, Austria) in plate–plate configuration with a 1 mm gap was used. The samples were dried overnight at 80 °C and tested in an angular frequency range of 1−500 s^−1^ under strain of 1%, which was in the linear viscoelastic (LVE) range, as proven by a preliminary amplitude sweep. An evaluation of zero shear viscosity (η_0_) was possible due to the rheological measurements in oscillatory mode and the calculations performed with Rheoplus 32 software V.3.40 (Anton Paar GmbH, Graz, Germany). A temperature of 230 °C was applied during the test to make sure that no polymer degradation would take place and violate the results. 

#### 2.2.5. Fourier Transform Infrared (FT-IR) Spectoscopy

Fourier transform infrared (FT-IR) spectroscopy was carried out using a FT/IR-4600 spectrometer (Jasco Europe S.R.L., Cremella, Italy). The spectra were collected at room temperature over a wavelength range of 4000−400 cm^−1^, with a resolution of 4 cm^−1^, by averaging 40 scans. Spectroscopic data were evaluated using the software Spectra Manager (ver. 2, Jasco, Easton, MD, US).

#### 2.2.6. Thermal Differential Scanning Calorimetry (DSC)

The melting and crystallization behavior of the R-PE/R-PA blends grafted by PE-g-MAH was studied using a DSC 204 F1 Phoenix (Netzsch GmbH, Selb, Germany), operated with a protective atmosphere (nitrogen flow of 20 mL/min). Samples of about 8 mg were first heated from room temperature to 270 °C at a rate of 10 °C/min and kept at that temperature for 5 min, followed by cooling to 20 °C at a rate of 10 °C/min to eliminate the thermal and mechanical history of polymers. The samples were subsequently reheated to 270 °C to determine the enthalpy of melting and cooled at the same rate. The thermal positions of the exothermic and endothermic peaks were taken as crystallization and melting temperatures (T_cr_ and T_m_). The crystallization and melting enthalpy (Δ*H_m_*) were evaluated from the exothermic and endothermic peak areas. The degree of crystallinity (*X_c_*) of the polyethylene, a major component of the R-PE/R-PA blends, was determined using the following equation:

(2) $$X_{c}\left(\%\right)=\frac{\Delta H_{m}}{\varphi_{PE}\Delta H_{m}^{0}}\;\cdot \;100$$

where Δ*H_m_* is the melting enthalpy of the sample (J/g), $\Delta H_{m}^{0}$ = 293 J/g is the melting enthalpy of 100% crystalline polyethylene [35], and *φ_PE_* is the R-PE content in the samples.

#### 2.2.7. Thermogravimetry (TGA)

To check the composition of the blends, thermogravimetric analysis (TGA) was carried out using a TG 209 F1 (Netzsch GmbH, Selb, Germany), operated with a protective nitrogen atmosphere with a flow rate of 60 mL/min. Samples (10 ± 0.2 mg) were placed in a ceramic crucible and heated from room temperature to 800 °C at a rate of 10 °C/min. The thermal properties of the blends were determined from TGA and DTG measurements as the temperature at which the mass loss was 5% (T_5%_) and the temperature of the maximum mass loss rate (T_max_).

#### 2.2.8. Mechanical Testing

The mechanical properties of the R-PE/R-PA blends were determined by tensile tests performed using a Zwick/Roell Z010 universal testing machine (Zwick GmbH & Co. KG, Ulm, Germany), together with the testXpert II program. The sample size was 110 mm × 6 mm × 4 mm, according to the ISO 527-2:2012 standard [36]. Tensile characteristics were measured at room temperature with a crosshead speed of 50 mm/min. Young’s modulus (E), ultimate tensile strength (σ_M_), tensile stress at break (σ_B_), and elongation at break (ε_B_) were evaluated from the tensile stress–strain curves. The reported data are averages of the results of 10 specimens. The hardness of samples was also measured using a Shore hardness tester (HBD 100–0, Sauter GmbH, Balingen, Germany) according to the PN-EN ISO 868:2005 [37]. The hardness was indicative of an average penetration value (Shore degrees on the D scale) based on 10 readings from tests.

## 3. Results

### 3.1. Morphological Aspects

**Figure 1:**
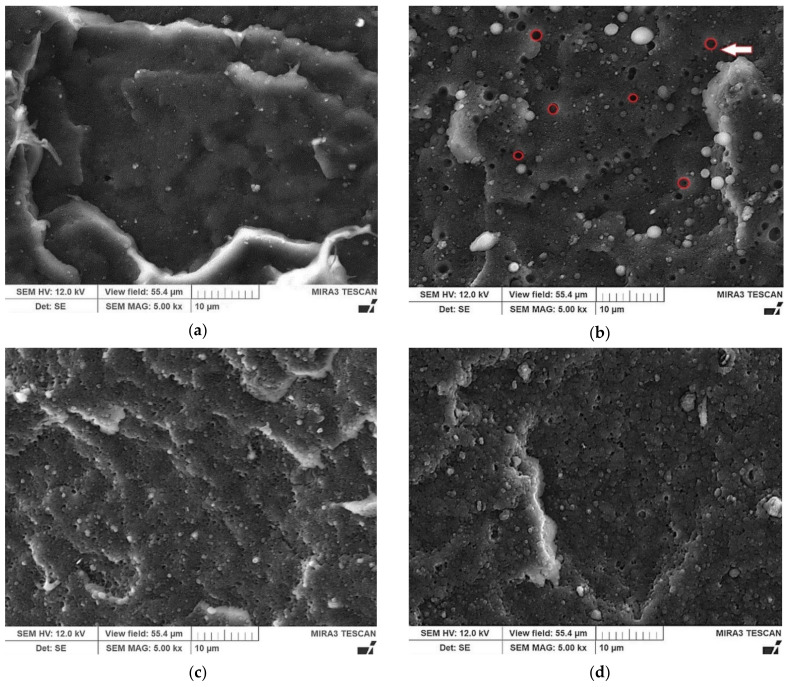
SEM micrographs of fractured surface of (**a**) R-PE and R-PE/R-PA (**b**) without and (**c**) with compatibilizer R-PE/R-PA/MAH1 and (**d**) R-PE/R-PA/MAH3; magnification 5000×.

The morphology and compatibility of R-PE/R-PA blends with and without PE-g-MAH were investigated by scanning electron microscopy (SEM). The results are given in Figure 1. 

Figure 1 shows SEM images of the fractured surfaces of R-PE, R-PE/R-PA binary blend, and R-PE/R-PA/MAH blends. This shows that PE and PA regranulates are incompatible by melt blending (Figure 1b). A two-phase morphology is observed on the fractured surface of the R-PE/R-PA blend. Polyamide disperses in a spherical shape in the continuous phase of polyethylene matrix. Figure 1b shows the “holes” (red circles) formed during fracture, which means that the weakly bound PA dispersed phase was “pulled out” from the polyethylene matrix. A similar effect in the PET/PA6 blend was observed by Lin et al. [38]. Because there is no adhesion between the R-PA phase and the R-PE polymer matrix, the cavities of the R-PA particles are clear and smooth. Adding a small quantity of compatibilizer to the blend resulted in changes in the morphology of the R-PE/R-PA blends (Figure 1c,d). 

Figure 1c,d shows SEM micrographs of R-PE/R-PA blends after compatibilization by different PE-g-MAH content. By adding 1 wt.% PE-g-MAH, the size of the domain phase decreased, and the distribution became more uniform. It can be seen that the dimensions of dispersed PA domains decreased (from 2 ± 0.03 μm to less than 0.5 ± 0.05 μm). Based on the blend morphology, it can be seen that 1 wt.% PE-g-MAH added to the R-PE/R-PA waste blend increased the interfacial interactions and compatibility between polyethylene and polyamide 6, resulting in decreased polyamide particle size. Similar results were obtained for PA6/PE blends-clay nanocomposites by Scaffaro [39] and found that in the blends PA6/HDPE with an ethylene-co-acrylic acid copolymer (EAA) the dimension of the particles of the PA6 dispersed phase is smaller and the interfacial adhesion better [39].

### 3.2. Density and Melt Mass-Flow Rate (MFR) Analysis 

The results of density and melt flow rate, the basic processing property of polymers, are presented in Table 2. MFR enables indirect assessment of the melt behavior of blends (viscosity), and the selection of technologies and parameters for further processing [10].

As shown in Table 2, for the R-PE/R-PA blend with 1 wt.% compatibilizer, the density increased, and the density did not change for the blend with 3 wt.% PE-g-MAH content. This may be due to the “better packing” of R-PA in a R-PE matrix as a result of better dispersion of the components involving compatibilizer. The compatibilizing agent improved the adhesion between the R-PA and R-PE phases, which decreased the gaps and free spaces at the interface, thus increasing the density of the blend. 

The analysis of processing properties based on MFR shows that for the blend of R-PE/R-PA waste with 1 wt.% compatibilizer, a decrease in MFR occurred, i.e., an increase in the viscosity of the mixture, compared to polyethylene. This was due to the change in intermolecular interactions resulting from the formation of block polymers, which was a result of chemical reactions, as confirmed in the literature [19].

### 3.3. Water Absorption Analysis 

The changes of water absorbency for polyamide, polyethylene, and their blends with grafted PE-*g*-MAH are shown in Table 3. 

It can be seen from Table 3 that water absorptivity increased with addition of R-PA. This is due to the polar groups of PA, which absorb significant amounts of water, and PE and PE-g-MAH have little ability to absorb water [40]. However, the presence of moisture can significantly reduce the strength and dimensional stability of the products [41]. In the case of blends, water absorption decreased with the addition of PE-g-MAH compared to polyamide. For blends containing 1 and 3 wt.%, the reduction in water absorptivity was similar to the blend without PE-g-MAH. This is due to the homogeneity of the structure under the influence of the compatibilizing agent, which reduces the interactions of the strongly bound water molecules in the polyamide with polar amide groups. Hence, the less polar the polyamide particle, the lower the water absorption of the R-PE/R-PA blend [41], and for this reason the dimensional stability of products made with this blend is much higher.

### 3.4. Viscosity Evaluation

**Figure 2:**
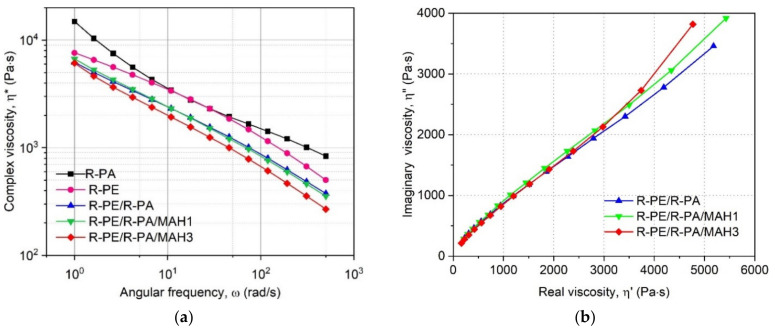
(**a**) Complex viscosity (η*) as a function of angular frequency (ω) and (**b**) imaginary part of viscosity (η”) as a function of real part of viscosity (η’) of studied materials at 230 °C.

The flow curve (complex viscosity η* as a function of angular frequency ω) of the studied materials at 230 °C is presented in Figure 2. 

As shown in Figure 2a, all tested samples presented shear-thinning behavior in the applied range of angular viscosity, which is rather typical for these materials [29]. Even though the course of the η* vs. ω curves obtained for both polymers and their blends is similar, some differences can be seen. The most interesting behavior is presented by the R-PA sample: the slope of the curve becomes steeper in the lower frequency range. It can be presumed that this material does not approach the Newtonian plateau. Very similar results were obtained by Bai et al. for polyamide 12 at 230 °C [42]. This behavior can be explained by the fact that at higher shear rates, the polymeric chains move relative to each other more easily and their entanglement decreases. The complex viscosity of the unfilled R-PE also decreases with the angular frequency, but the slope of the curve becomes less steep in the lower ω range, which results from the different molecular structures of the two polymers. 

The R-PE/R-PA blend can be characterized by similar rheological properties as its components: the curve presented in Figure 2a is parallel to the one of R-PA but shifted toward lower viscosity values. This result can indicate the low miscibility of both polymers; the presence of polyamide macromolecules does not limit the possibility of movement of polyethylene chains and vice versa. 

The addition of 1 wt.% MAH did not significantly change the run of the η* vs. ω curve, except for a small increase in the lower angular frequency range. This may indicate a slight enhancement of interactions between phases [43,44]. In the case of the R-PE/R-PA/MAH-3 sample, decreased viscosity can be observed, which is probably caused by the lower content of highly viscous polyamide in the modified blend and a higher percentage of molten maleic anhydrite, which flows easily and acts as a plasticizer. Similar results were obtained by Bai and Dou for polypropylene/polylactide blends modified by maleic anhydrite-grafted polypropylene [42]. It can be concluded that the presence of the modifier should not cause difficulties related to the flow of the polymers, but rather will enhance the processability of the blends. 

Cole–Cole plots representing imaginary viscosity η” as a function of real viscosity η’ are shown in Figure 2b. Such plots are commonly used to evaluate the miscibility of polymeric blends. Good compatibility of the studied components is indicated by the semicircular shape of the plot [45]. As can be seen in Figure 1b, the plot of the R-PE/R-PA blend is linear rather than semicircular, which confirms that the two components create a non-homogeneous mixture. Adding the compatibilizing agent does not result in a more circular shape of the plot. Due to the complex composition and morphology of the recycled materials, the Cole–Cole plot may not be the best method to evaluate the miscibility of the blends.

### 3.5. Fourier Transform Infrared (FT-IR) Analysis

**Figure 3:**
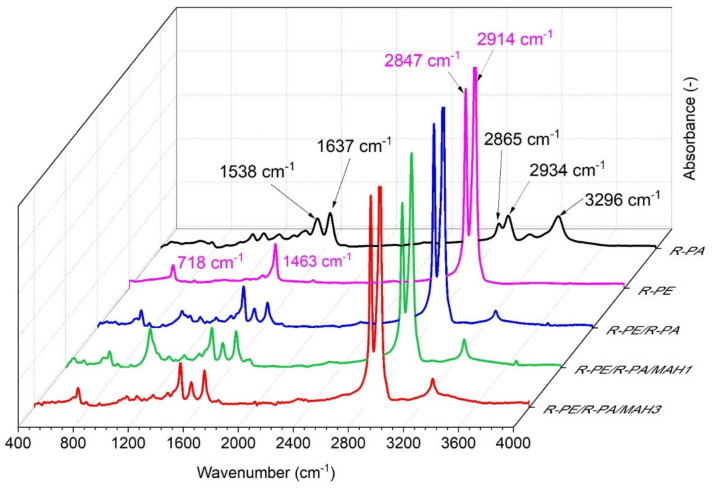
FT-IR spectra of R-PE, R-PA, R-PE/R-PA binary blend, and R-PE/R-PA blends grafted by PE-g-MAH.

Fourier transform infrared (FT-IR) spectroscopy was used to identify peak shifts between the two polymers, to detect whether the type of interaction between the materials was strong or weak [46]. Figure 3 shows the FT-IR spectra of pure materials R-PE and R-PA, the R-PE/R-PA blend, and the R-PE/R-PA blend with the addition of PE-g-MAH compatibilizer. 

As shown in Figure 3, pure R-PE shows characteristic strong peaks at 2914 and 2847 cm^–1^ associated with the asymmetric and symmetric aliphatic –CH_2_ stretching vibration of carbon–hydrogen bonds [47,48] and bending vibrations at 1463 and 718 cm^–1^. The absorption at 1463 cm^–1^ corresponds to the stretching vibration attributed to the CH_2_ bond (–CH_2_ scissoring) and the band at 720 cm^−1^ (–C–C rocking) refers to the stretching vibration of the carbon–carbon bond [49]. The polyamide spectrum presents bands of the ester group, C=O asymmetrical stretching of the imide group at 1738 cm^−1^, amide I (C=O stretching) at 1637 cm^–1^ amide II (C–N stretching and N–H bending of hydrogen-bonded N–H groups at 1538 cm^−1^ [50]. The band at 3296 cm^–1^ is associated with hydrogen-bonded N–H stretching (amide A). The two strong bands at 2934 and 2865 cm^−1^ correspond to the asymmetric with respect to the symmetric C–H stretching vibrations [47]. R-PE/R-PA blends show N–H stretching at 2915 cm^–1^, C=O stretching of the imide group at 1740 cm^−1^, C=O stretching of the amide group at 1633 cm^–1^, and N–H bonding and C–N stretching of amide II at 1533 cm^−1^ [46]. It can be seen that all R-PE/R-PA blends with PE-g-MAH compatibilizer show similar peaks to the R-PE/R-PA blend, except for the region around 1000 cm^–1^, where the PE-g-MAH1 blend shows a stronger signal. No additional peaks of ester groups derived from maleic anhydride were observed. According to Tjong et al. [51], this is due to degradation of the MAH group grafted into –COOH during the melting process.

### 3.6. Thermal Differential Scanning Calorimetry (DSC) Results

**Figure 4:**
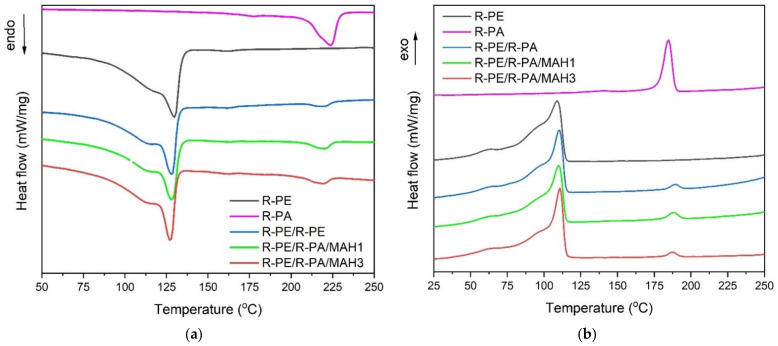
DSC (**a**) melting and (**b**) crystallization curves of R-PE, R-PA, R-PE/R-PA binary blend, and R-PE/R-PA blends grafted by PE-g-MAH.

Heating and cooling DSC thermograms of the investigated samples are presented in Figure 4. The melting and crystallization temperatures and the melting enthalpy Δ*H_m_* for R-PE, R-PE/R-PA, and R-PE/R-PA/MAH blends are summarized in Table 4.

As can be seen in Figure 4, all blends reveal two melting peaks T_m1_ and T_m2_, characteristic of immiscible polymer mixtures with two phases [52]. The first one, T_m1_ at about 125 °C, and the second, T_m2_ at about 224 °C, correspond to the melting of polyethylene (R-PE) with respect to polyamide 6 (R-PA). The presence of these two values indicates the heterogeneous structure of the blends [19]. From these data, a slightly higher melting temperature (T_m1_) of R-PE/R-PE at 3 wt.% PE-g-MAH loading was observed, which indicates the interfacial interaction between the blend in the presence of the compatibilizer. Moreover, two separate crystallization peaks (T_cr1_ and T_cr2_) during the DSC cooling run (see Figure 4) were observed. The temperature of 184.6 °C corresponds to polyamide crystallization and about 109.0 °C to polyethylene crystallization. The presence of two crystallization peaks is also confirmation of the heterogeneous structure of the R-PE/R-PA/MAH blends. The polyethylene crystallization temperature (~ 109 °C) the slightly increased, up to about 113 °C with the PE-g-MAH concentration, in particular for 3 wt.% of the R-PE/R-PA/MAH3 blend, indicating that the PE-g-MAH may act as a nucleating agent that affects the polyethylene matrix. As shown in Table 4, a distinct increase in the degree of crystallization (X_c_), as well as the heat of melting for R-PE/R-PA blends were noted. The X_c_ value, for the R-PE/R-PA/MAH1 blend increased from 43% for the R-PE/R-PA binary blend, up to about 47% for the R-PE/PA blend modified with 1 wt.%. of PE-g-MAH, indicating the chemical interactions between the PE-g-MAH and polyamide, which implied the molecular chain mobility of the R-PA that is needed for crystallization, resulting in an increase in polyamide crystallinity. A similar trend was observed by Zhang et al. [53], who investigated the effect of multiwall carbon nanotubes (MWCNTs) on the crystallization of polyamide in a blend with polypropylene. They found that the addition of MWCNTs to a R-PA/R-PP blend resulted in increased crystallinity of the PA phase. Similar results were reported by Yousfi et al. [54] in the case of PA/PP/PP-g-MAH blends.

### 3.7. Thermogravimetric Analysis (TGA) Results

**Figure 5:**
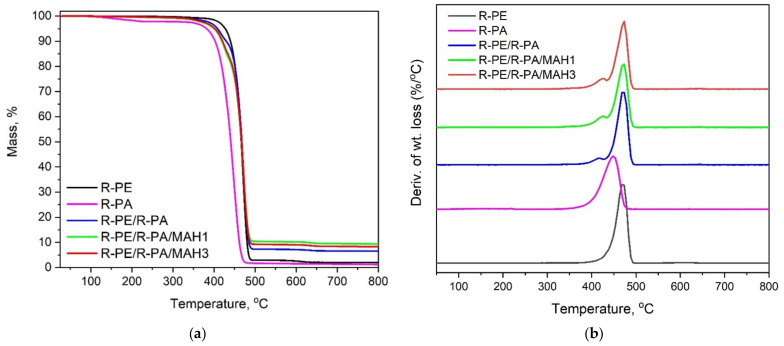
(**a**) TGA and (**b**) DTG curves of R-PE, R-PA, R-PE/R-PA binary blend, and R-PE/R-PA blends grafted by PE-g-MAH (heating rate: 10 °C/min; nitrogen atmosphere).

The characteristic TGA curve and its first derivative differential of thermogravimetry (DTG) curve are shown in Figure 5. Thermogravimetric measurements are usually applied to detect the composition of blends, copolymers, and composites and to characterize the degradation process. 

In our case, the temperature T_5%_ and T_50%_ of R-PE/R-PA/PE-g-MAH samples, the maximum temperature of decomposition of the blends T_max_, and the residues at 800 °C are taken into account (see Table 5).

From Table 5, we can find that the polyethene (R-PE) and polyamide (R-PA) undergo a single-step degradation process with onset at 470 and 450 °C, respectively. In the case of R-PE/R-PA blends, we can see a two-step decomposition process with a decomposition temperature starting at about 430 °C, corresponding to the degradation of the PA phase, and the second region of the main decomposition stage at a temperature of 470 °C. Additionally, it can be seen in Table 5 that the char residue at 800 °C for R-PE/R-PA blends grafted with PE-g-MAH is about 9 mass %, which means it is higher compared to R-PE/R-PA binary blend and polyethylene matrix (by about 2%), indicating that the compatibilized R-PE/R-PA/MAH blends have better char-forming ability than polymer matrix.

From Figure 5a we can see that the decomposition temperature (T_5%_) increases when PE-g-MAH is added to the PE/PA blend. When 3 wt.% of PE-g-MAH is added, the decomposition temperature (as measured at the point of 5% mass loss) increases from 401 to about 408 °C. This means that the R-PE/R-PA/MAH blend has the highest thermal stability compared with polyethylene and polyamide. This may be due to the good dispersion of the polyamide in the polyethylene matrix with the participation of the compatibilizer. According to Zhai, the reason for this may be that the thermal properties of PE-g-MAH are much better than those of polyethylene [55]. The result is consistent with what was found by Yousi et al. [54] in case of talc-filled PP/PA nanocomposites. The authors noted that the addition of talc fillers induced a significant decrease in the size of the PA6 domains, which is related to the higher thermal stability of the PP/PA blends [54]. A similar effect was observed by Araújo et al. in a mixture of polyamide with high-density polyethylene and the compatibilizer [56]. 

### 3.8. Tensile Testing and Hardness Results

**Figure 6:**
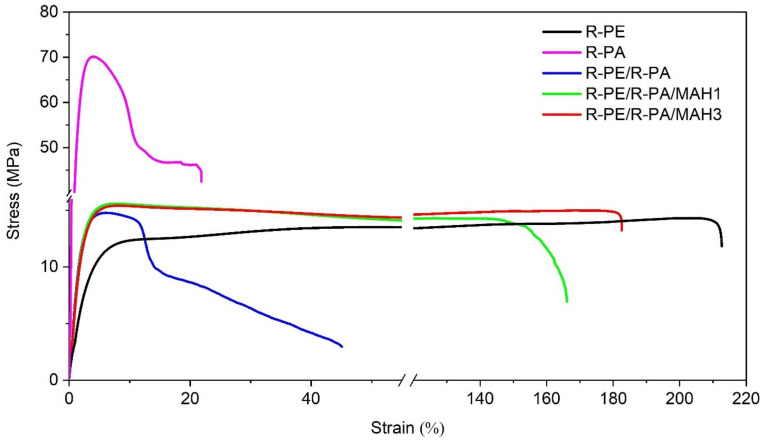
Stress–strain curves of R-PE, R-PA, and R-PE/R-PA blend with varying compatibilizer content.

Figure 6 presents the tensile stress–strain curves for R-PA, R-PE, and R-PE/R-PA blends based on the procedure described in the previous section. Regranulated R-PE/R-PA blends grafted with 1 and 3 wt.% PE-g-MAH as compatibilizer were investigated. We can see that the R-PE curves are ductile and those of R-PE with R-PA blend based on wastes are rigid and brittle, like polyamide waste. However, significant differences can be seen in the curves for the PE-g-MAH modified blends. The strain–stress curves of the R-PE/R-PE blends show a significant increase in elongation at break compared to polyethylene/polyamide blend.

The effects of different PE-g-MAH content on the Young’s modulus (E), ultimate tensile strength (σ_M_), tensile stress at break (σ_B_), elongation at break (ε_B_), and Shore’a hardness of all samples are summarized in Table 6. 

The results show that ultimate tensile strength for the R-PE/R-PA blends increased with the addition of PE-g-MAH as a compatibilizer. Tensile stress at break for R-PE/R-PA blends increased for samples with MAH compatibilizer, indicating that a small amount of 1 wt.% PE-g-MAH leads to better interfacial adhesion between the blend components.

As shown in Figure 6, the tensile strength at break of R-PE decreased when recycled polyamide was added, and then increased with the addition of PE-g-MAH. In the blend that contained 3 wt.% MAH, Young’s modulus and tensile strength increased by 190 and 19%, respectively, compared to recycled polyethylene. The result shows that the hardness of samples increased for the blends with R-PE/R-PA and PE-g-MAH. 

The increased strength and hardness of R-PE/R-PA blends with the addition of a compatibilizer in relation to the two-component mixture may indicate the compatibilizing effect of PE-g-MAH. It can be concluded that during the reactive extrusion process, block copolymers can form at the phase boundary, causing the formation of strong interfacial bonds between PE-g-MAH groups and end groups of polyamide 6 [57].

Similar results were presented by Hamid et al. [46], who noted that incorporating HDPE-g-MAH into blends improved hardness as a result of increased interfacial adhesion in the blends. As seen in Table 6, for the R-PE/R-PA blend with PE-g-MAH, we observed an increase in both tensile strength and elongation at break. The elongation at break of the R-PE/R-PA/MAH3 compatibilized blends increased from 48 to 186%, which corresponds to an increase of approximately 300%. The increase in tensile stress at break of the R-PE/R-PA/PE-g-MAH blend can be attributed to the increased degree of crystallinity, due to the increased miscibility of thermodynamic non-miscibility of polyethylene and polyamide mixture wastes. There is good agreement with the calorimetric results. 

The results show a significant increase of Young’s modulus for the R-PE blend with PA, which corresponds to an increase in stiffness to about 100% compared to the recycled polyethylene. The higher tensile modulus values of the R-PE/R-PA regranulate shows that the addition of the PE-g-MAH by extrusion to the polyethylene/polyamide binary blends improved partial miscibility and compatibility of these blends. A similar behavior in reactive compatibilization polyamide 6/olefin block copolymer blends has been reported by Lin, et al. [38].

## 4. Conclusions

The investigations carried showed a significant influence of PE g-MAH on morphology and mechanical properties of the R-PE/R-PA blends. In this study, the R-PE/R-PA blends that contained PE-g-MAH was prepared by reactive extrusion in a twin-screw extruder. It was found that the addition of PE-g-MAH allows for an advantage combination of immiscible polymers come from waste. Thus, it was proven that this connection is possible, and from the obtained blends, it is possible to produce regranulates for industrial applications. 

A morphological study showed that the PE-g-MAH significantly improved the structure of the R-PE/RPA/MAH blend compared to R-PE/R-PA binary blend, resulting in reduction dimension of R-PA domains size of the disperse phases and better interfacial adhesion. SEM presented that adding a small (1 wt.%) amount of PE-g-MAH compatibilizer to the R-PE/R-PA waste blend increases the interfacial interactions of this composition, thus ensuring its structural coherence.

R-PE/R-PA blends containing 1 wt.% compatibilizer showed reduced water absorption compared to the polyamide, due to the homogeneity of the R-PE/R-PA blend grafted by PE-g-MAH structure under the influence of the compatibilizing agent, which reduces the interactions of the strongly bound water molecules in the polyamide with polar amide groups. Viscosity analysis showed that in the case of blends made of R-PE/R-PA waste with the addition of a compatibilizer, there is a slight decrease in the viscosity of the blend in relation to R-PE or R-PE/R-PA binary blend, due to the lower viscosity of PE-g-MAH compared with polyethylene and polyamide. However, the decreased MFR shows that the influence of the compatibilizing agent on the rheological properties of the blend depends on the applied shearing conditions. It can also be concluded that the application of PE-g-MAH should not cause problems during processing of the blends. The results of the mechanical properties show a significant increase in Young’s modulus for the R-PE blend with R-PA grafted by PE-g-MAH, which corresponds to an increase of about 100% compared to recycled polyethylene. The higher tensile properties in R-PE/R-PA/MAH were related to an increase in crystalline structure. This demonstrates its improved stiffness and strength when considering using this material for films with increased strength and resistance to destruction, due to the increased degree of crystallinity of R-PE/R-PA with compatibilizer compared to polyethylene matrix. 

The proposed method of waste management with defined characteristics and structure to produce regranulates based on unmixed polymers of polyethylene and polyamide with the addition of a compatibilizer may be attractive in terms of application in the packaging industry for the production of films. In particular, the use of recycled polyethylene and polyamide can be a useful technique for processing regranulates based on immiscible polymers, creating a sustainable solution for an environmental problem. This study proves that R-PE/R-PA regranulates are waste plastics that can be combined despite their incompatibility to create materials for further applications, which is in line with the circular economy principle.

## Figures and Tables

**Table 1:** Sample codes and polymer blend compositions.

Sample Code	Composition R-PE/R-PA/PE g-MAH [wt.%]
R-PA	0/100/0
R-PE	100/0/0
R-PE/R-PA	80/20/0
R-PE/R-PA/MAH1	80/19/1
R-PE/R-PA/MAH3	80/17/3

**Table 2:** Results of density and melt flow rate (MFR) of samples.

Sample	Density [g/cm^3^]	MFR _(2.16 kg, 230 °C)_ [g/10 min]
R-PA	1.181 ± 0.110	9.560 ± 0.174
R-PE	0.949 ± 0.006	1.030 ± 0.095
R-PE/R-PA	1.008 ± 0.014	1.535 ± 0.010
R-PE/R-PA/MAH1	1.012 ± 0.039	1.330 ± 0.062
R-PE/R-PA/MAH3	1.008 ± 0.019	1.237 ± 0.467

**Table 3:** Water absorption test results of samples.

Sample	Water Absorption [%]
R-PA	2.01 ± 0.11
R-PE	0.00 ± 0.00
R-PE/R-PA	0.56 ± 0.16
R-PE/R-PA/MAH1	0.14 ± 0.04
R-PE/R-PA/MAH3	0.22 ± 0.01

**Table 4:** DSC data of R-PE, R-PA, R-PE/R-PA, and R-PE/R-PA/MAH blends (second heating at 10 °C/min); X_c_ (%) is the crystallinity degree of polyethylene phase in the blend.

Sample	T_m1_ [°C]	ΔH_m1_ [J/g]	T_m2_ [°C]	T_cr1_ [°C]	T_cr2_ [°C]	X_c_ [%]
R-PA	223.8	−29.8	-	-	184.6	15.7 ± 0.5
R-PE	125.0	−135.4	-	109.4	-	46.2 ± 1.1
R-PE/R-PA	126.0	−99.8	218.9	111.3	189.6	42.6 ± 0.7
R-PE/R-PA/MAH1	126.7	−110.9	219.3	111.5	189.4	47.3 ± 0.6
R-PE/R-PA/MAH3	127.0	−102.1	220.1	112.6	190.1	44.5 ± 1.1

**Table 5:** Temperature T_5%_ and T_50%_ at mass loss of 5 wt.% and 50 wt.%, respectively, maximum peak temperature decomposition, and residual mass at 800 °C for R-PA, R-PE, and their blends.

Sample	T_5%_ [°C]	T_50%_ [°C]	Decomposition Temperature (Peak) [°C]	DTG [%/^o^C]	Residue at 800 °C [wt.%]
R-PA	382.0	440.5	448.7	−20.7	1.2
R-PE	439.0	466.0	470.3	−31.4	2.0
R-PE/R-PA	408.4	467.4	467.6	−28.5	6.5
R-PE/R-PA/MAH1	400.0	466.5	473.0	−24.9	9.4
R-PE/R-PA/MAH3	402.0	466.8	475.0	−28.2	8.3

**Table 6:** Results of mechanical testing.

Sample Code	Young’s Modulus, E [MPa]	Ultimate Tensile Strength, σ_M_ [MPa]	Tensile Stress at Break, σ_B_ [MPa]	Elongation at Break, ε_B_ [%]	Hardness Shore’a [^o^Sh]
R-PA	1130 ± 15	70.4 ± 0.3	42.6 ± 1.1	25 ± 12	80.3 ± 0.5
R-PE	254 ± 17	11.9 ± 0.2	10.2 ± 1.0	210 ± 11	54.2 ± 0.3
R-PE/R-PA	371 ± 12	19.7 ± 0.1	2.9 ± 0.1	48 ± 18	56.1± 0.2
R-PE/R-PA/MAH1	456 ± 16	20.5 ± 0.4	18.5 ± 1.7	165 ± 15	57.2 ± 0.1
R-PE/R-PA/MAH3	474 ± 11	21.7 ± 0.2	20.1 ± 0.6	187 ± 23	61.3 ± 0.3

## Data Availability

The data presented in this study are available on request from the corresponding author.
